# Necrotic Ulcers Secondary to Apomorphine Infusion

**DOI:** 10.5334/tohm.648

**Published:** 2021-12-27

**Authors:** Yudy Llamas-Osorio, Caitriona McLoughlin, Michael Maguire, Tim Lynch

**Affiliations:** 1 Department of Neurology, Dublin Neurological Institute at the Mater Misericordiae University Hospital, Dublin, IE; 2 Dermatology Department, Mater Misericordiae University Hospital, Dublin 7, IE

**Keywords:** Apomorphine, ulcers

## Abstract

**Background:**

Apomorphine is a potent dopamine agonist used in the treatment of advanced and fluctuating Parkinson’s Disease. However the need for its subcutaneous infusion can lead to skin reactions.

**Phenomenology Shown:**

We illustrate necrotic ulcers at infusion sites as a rare event during continuous subcutaneous apomorphine infusion.

**Educational value:**

This case demonstrates the rare adverse event of necrotic ulcers in a patient with long term apomorphine infusion.

We describe an 81 year-old man with a 17 year history of levodopa responsive Parkinson’s Disease (PD), treated with Continuous Subcutaneous Apomorphine Infusion (CSAI) at rate of 5 mg/hour × 12 hours daily for 3 years.

Other medications include L-Dopa 1000 mg daily, Rasagiline 1 mg daily, Mirtazapine 7.5 mg nocte, Clonazepam 1 mg nocte and Apixaban 2.5 mg twice daily. In the last month he developed multiple subcutaneous nodules and changing the injections site was decided. He then experienced ulcerative lesions on his thighs and shoulders 24 hours post infusion at each injection site. (***Figures 1 and 2***)

**Figure 1 and 2:**
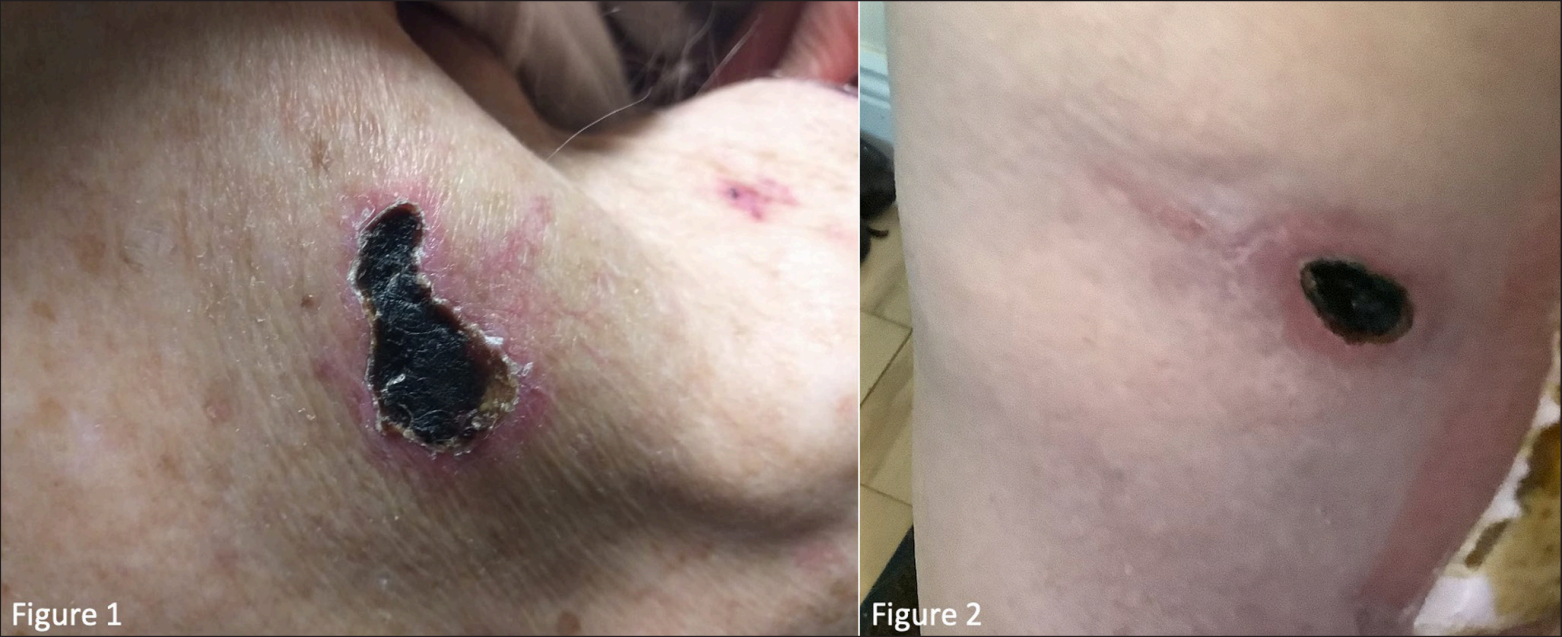
Are showing injection site reactions with skin necrosis and overlying eschar at his right shoulder and thigh.

Subcutaneous administration of apomorphine is generally well tolerated. Site reactions, such as nodules, are mild and temporary. However in rare cases these lesions can become necrotizing. Another report noted similar results in a young patient with longstanding PD, which resolved over one month following discontinuation [1,2].

An expert consensus recommendation to manage apomorphine therapy-related skin nodules was published, because up to 92% of patients on CSAI can experience skin problems [3].

In our patient the skin lesions did not improve despite changing infusion sites and preparation. His skin started to improve four weeks after discontinuation of CSAI.
